# LPS Modulates the Expression of Iron-Related Immune Genes in Two Antarctic Notothenoids

**DOI:** 10.3389/fphys.2020.00102

**Published:** 2020-02-14

**Authors:** Danixa Pamela Martínez, Carmen Sousa, Ricardo Oyarzún, Juan Pablo Pontigo, Adelino V. M. Canario, Deborah Mary Power, Luis Vargas-Chacoff, Pedro Miguel Guerreiro

**Affiliations:** ^1^Instituto de Ciencias Marinas y Limnológicas, Universidad Austral de Chile, Valdivia, Chile; ^2^Centro Fondap de Investigación de Altas Latitudes (IDEAL), Universidad Austral de Chile, Valdivia, Chile; ^3^Centro de Ciências do Mar (CCMAR), Universidade do Algarve, Faro, Portugal; ^4^Escuela de Graduados, Programa de Doctorado en Ciencias de la Acuicultura, Universidad Austral de Chile, Puerto Montt, Chile

**Keywords:** *Notothenia coriiceps*, *Notothenia rossii*, iron metabolism, Antarctic fish, nutritional immunity

## Abstract

The non-specific immunity can induce iron deprivation as a defense mechanism against potential bacterial pathogens, but little information is available as to its role in Antarctic fish. In this study the response of iron metabolism related genes was evaluated in liver and head kidney of the Antarctic notothenoids *Notothenia coriiceps* and *Notothenia rossii* 7 days after lipopolysaccharide (LPS) injection. Average plasma Fe^2+^ concentration was unaffected by treatment in any of the species. The gene expression response to LPS varied between tissues and species, being stronger in *N. coriiceps* and more prominent in the head kidney than liver. The reaction to LPS was marked by increased individual variability in most genes analyzed, even when the change in expression was not statistically significant, suggesting different individual sensitivity and coping responses in these wild fish. We found that iron related genes had an attenuated and homogenous response to LPS but there was no detectable relationship between plasma Fe^2+^ and gene expression. However, overall in both tissues and species LPS exposure set a multilevel response that concur to promote intracellular accumulation of iron, an indication that Antarctic Notothenoids use innate nutritional immunity as a resistance mechanism against pathogens.

## Introduction

The vertebrates, including fishes, have developed innate and an adaptive immune systems composed both cellular and humoral components to respond to pathogens (Barandica and Tort, 2008). Both systems can be communicated through soluble mediators known as cytokines that regulate the magnitude of the immune response, controlling pathogen growth, promoting inflammation and triggering the adaptive immune response (Sompayrac, 2012). The main organs with immunological function in fish are the thymus, spleen, liver, head kidney and mucosa-associated lymphoid tissue (MALT). They can be grouped into primary (thymus and head kidney) and secondary (spleen, liver, and MALT) immune organs according to their participation in the production, maturation, activation and proliferation of immune components (Soulliere and Dixon, 2017).

During inflammation, the innate immune system can induce several antimicrobial mechanisms, including the depletion of iron available to the pathogen at the systemic and cellular levels (Johnson and Wessling-Resnick, 2012). This defense mechanism, known as nutritional immunity, consists of the removal of iron from the circulation and its subsequent sequestration inside the cell (Collins, 2008). Proinflammatory cytokines such as IL-6 stimulate the transcription of hepcidin, triggering and potentiating the hypoferric response of inflammation (Nemeth et al., 2004a). Several bacterial pathogens obtain the iron they need for their survival and replication from an external source (Ratledge and Dover, 2000; Pulgar et al., 2015; Bethke et al., 2016, 2018; Calquín et al., 2018). For this reason, eukaryotic organisms must effectively control iron homeostasis, through the regulation of the proteins involved in its metabolism (Skaar, 2010).

In fish, iron is mainly taken up by the intestine (Bury and Martin, 2003) where it can enter as heme iron, Fe^2+^ and Fe^3+^ (Bury and Martin, 2003). Duodenal cytochrome B is necessary to convert Fe^3+^ to Fe^2+^ (Bury and Martin, 2003). The pool of Fe^2+^ interacts with the divalent metal transporter present on the apical side of the enterocytes (Gunshin et al., 1997; Andrews, 1999). Once inside the cell, the iron can be stored in the cytoplasmic ferritin (the portion of iron that is not for immediate use) (Torti and Torti, 2002), directed toward the mitochondria (for biosynthesis of the Fe-S cluster and heme group) or used in other routes of iron metabolism (Hentze et al., 2004).

The exit of iron from the enterocytes is regulated by ferroportin (Donovan et al., 2000). However, hepcidin can induce the degradation of ferroportin, controlling the release of iron from the basolateral membrane into the bloodstream (Nemeth et al., 2004b). The export of iron also depends on the presence of a copper-associated oxidase, hephaestin in intestinal cells or of ceruloplasmin in non-intestinal cells (Harris et al., 1999; Vulpe et al., 1999). The ferroxidase allows the iron to be loaded on to the cytoplasmic transferrin to be directed to other tissues that need it (Hentze et al., 2004). Iron uptake can be then mediated by transferrin receptor 1 (TfRC1) that is expressed in all tissues (Cheng et al., 2004) or by transferrin receptor 2 (TfRC2), yet not described in fish, that in mammals is found in hepatocytes, duodenal crypt cells and erythroid cells, suggesting a more specialized role of this receptor in iron metabolism (Kawabata et al., 1999). In fish, such as *Salmo salar* and *Eleginops maclovinus*, the expression of the main proteins involved in iron metabolism appears to be modulated by the presence of live bacterial pathogens (Pulgar et al., 2015; Martínez et al., 2017a).

Research on Antarctic fish immune responses has mainly addressed the effect of thermal stress on the metabolism of carbohydrates and antioxidant defense system (Machado et al., 2014; Klein et al., 2017; Souza et al., 2018), or analyzed the level of transcriptional response of immune genes after stimulation with Poly I:C and with heat-killed cells, *E. coli* (Ahn et al., 2016) and *Psychrobacter* sp. (Buonocore et al., 2016). Furthermore, cDNAs of iron metabolism proteins have been cloned, e.g. *ceruloplasmin* (*Cp*), *transferrin* (*Tf*), *ferritin* (*Fth1*) and *divalent metal transporter 1* (*Dmt1*) (Scudiero et al., 2007, 2009), but no specific information is available on how they are regulated before an infectious process. Furthermore, there is no information on nutritional immunity associated with iron in Antarctic fish or if this type of immune response could be activated by the detection of pathogen-associated molecular patterns (PAMPs), which activate the immune system, but do not require iron. Therefore, the objective of the present study was to evaluate the transcriptional response of immune genes associated with iron in Antarctic Notothenoid fish (*Notothenia coriiceps* and *Notothenia rossii*) stimulated with a bacterial PAMPs (specifically LPS, a lipopolysaccharide that integrates the cell wall of gram-negative bacteria).

## Materials and Methods

### Sample Collection

*Notothenia coriiceps* (30 ± 2.4 cm of length and 384 ± 93 g of weight) and *N. rossii* (30 ± 4.2 cm of length and 312 ± 124.7 g of weight) were captured by hook-and-line from a boat, from 5–20 m deep in the waters near the Chinese Great Wall Station, at King George Island (GPS coordinates: 62°13′S, 58°58′W), in the Antarctic Peninsula, during late January 2017. Upon fishing fish were initially randomly placed in six 200 L flow-through plastic tanks for up to 3 days and then measured, weighed, tagged with opercular metal tags and allocated in groups with similar biomass and size distribution in the same flow-through system (two tanks per species; *n* = 7–8 per tank). Fish were anesthetized in phenoxyethanol (0.02 ml/L) before all experimental manipulations and left undisturbed for 1 week before the experiments to acclimate. At the beginning of the assay (day 0), fish were anesthetized as above and one group per species was injected intraperitoneally (IP) with saline (1.1% NaCl, 0.2 vol.% body weight) and acted as control, while the other group was IP-injected with LPS (1.5 mg/ml LPS in 1.1% NaCl, 0.2% vol body weight, to an effective dosage of 3 mg/kg). This procedure was repeated in day 2, when fish received a second injection of either saline (control) or LPS. At day 7 all fish were lethally anesthetized, blood was collected in heparinized syringes (ammonium salt heparin, 1000 U/ml, Sigma-Aldrich), and fish were sacrificed by cervical section to collect tissues. The head kidney and liver were dissected aseptically, immediately placed in RNAlater (Sigma-Aldrich), placed at 4°C for 24 h and then stored at −20°C. The blood was centrifuged at 10,000 *g* for 5 min at 4°C to obtain the plasma, which was subsequently stored at −80°C. Water parameters in tanks were monitored twice daily and were approximately constant throughout the experiment – temperature (2.1 ± 0.5°C), salinity (29 ± 0.5 ppt) and oxygen (10.5 ± 1.0 ppm). Two *N. coriiceps* died in the LPS group and no mortality was observed for *N. rossii*.

### Blood Plasma Iron

For determination of iron, 1 ml of 5% nitric acid (Sigma-Aldrich) was added to the blood plasma (110 μl), followed by sonication for 10 min and centrifugation at 16,000 *g* for 5 min at room temperature. The supernatant was removed to a new 15 ml falcon tube and the Fe^2+^ fraction was measured at 259.940 nm in an Agilent Microwave Plasma Atomic Emission Spectroscope (4200 MP-AES, Agilent Technologies). Only Fe^2+^ was measured due to limitations of plasma volume and of the spectrometer which could not measure the Fe^3+^ emission wavelength. The Agilent MP-AES Expert software was used to calculate concentration based on a 14 points standard curve (range 4.47E-05 mM–4.47E-08 mM) with automatic background subtraction provided by a blank (5% nitric acid), in accordance with the manufacturer’s instructions. The limits of detection (LOD) and limit of quantification (LOQ) were calculated from three and ten times the standard deviation of 15 consecutive blank measurements, respectively (Li et al., 2013). The values obtained were 3.77E-05 mM to LOD and 1.26E-04 mM to LOQ. The presence of interference was analyzed through spiking samples with 8.95E-04 mM (50 ppb) and 1.79E-03 mM (100 ppb) standards to the plasma samples and was found not to be significant. Recoveries were calculated from the same spiked plasma samples and estimated at 105%.

### RNA Processing and Quantification

Total RNA was extracted from 50 mg of tissue using Trizol (Ambion), following the manufacturer’s instructions. The RNA pellets were dissolved in diethylpyrocarbonate water (DEPC, Sigma-Aldrich) and stored at −80°C. RNA was quantified spectrophotometrically at 260 nm (NanoDrop Technologies) and quality was checked using electrophoresis in 1% agarose gels. Total RNA (2 μg) was used as a template for reverse transcription reactions to synthesize cDNA using MMLV reverse transcriptase (Promega) and the oligo-dT primer (Invitrogen).

The cDNAs were diluted to 100 ng (2 μL) and used as a template for quantitative polymerase chain reaction (qPCR) of *ferritin heavy chain* (*Fth1*), *ferritin middle chain* (*Fm*), *ferroportin* (*Fp*), *transferrin* (*Tf*), general identification as *transferrin receptor* (*TfRC*) on NCBI database, however, with higher similarity with *transferrin receptor type 1* (*TfRC1*) based in ENSEMBL and CCMAR Sea Genomics databases, general nomenclature as *hepcidin-like* (*Hamp*) identified on public NCBI database, however, has more similarity to *hepcidin type 2 variant 4* (*Hamp2*) based on CCMAR Sea Genomics database, *ceruloplasmin* (*Cp*), *interleukin-6 receptor alpha chain* (*IL-6R*α), *IL-6 receptor beta chain* (*IL-6R*β), and *18s ribosomal RNA* (*18S*). The primers for interleukin-6 were not considered because they did not work very well. These primers were designed based on *N. coriiceps* species because is the only genome partially published and available on public NCBI database^1^ and further confirmed by blast against of several known genome of fish species that are phylogenetically similar such as *Danio rerio*, *Oryzias melastigma*, *Oreochromis niloticus*, *Gasterosteus aculeatus*, *Tetraodon nigroviridis*, *Xiphophorus maculatus* by public ENSEMBL database^2^ and *Dicentrarchus labrax* and *Sparus aurata* fish species by public CCMAR Sea Genomics database^3^. The qPCR reaction was performed according to a published protocol (Martínez et al., 2017b), specifically: 95°C for 10 min, followed by 40 cycles at 90°C for 10 s, 60°C for 15 s, and 72°C for 15 s. Melting curve analysis of the amplified products was performed at the end of each PCR to confirm that only one PCR product was amplified and detected. Expression levels were analyzed using the comparative Ct method (2^–ΔΔCT^) (Livak and Schmittgen, 2001). Data were expressed as the fold change in gene expression normalized to an endogenous reference gene (housekeeping 18s) and relative to the untreated control. The primers used are listed in Table 1. PCR efficiencies were calculated according to the equation: *E* = 10 ^[–1/slope]^ (Rasmussen, 2001).

**TABLE 1:** qPCR primer pairs, product sizes and efficiency.

Primer	Nucleotide sequences (5′3′)	PCR product size	GenBank	*N. coriiceps*		*N. rossii*	
					
				Efficiency Liver (%)	Efficiency Head-kidney (%)	Efficiency Liver (%)	Efficiency Head-kidney (%)
Ferritin heavy chain (Fth1) Fw	AGTGGAGGCCCTTGAATGTGC	130pb	FM210467.1	97.4	109.4	95.8	94.2
Ferritin heavy chain (Fth1) Rv	GTCCAGGTAGTGAGTCTCGATGAA						
Ferritin middle chain (Fm) Fw	GGACACAGGATGCCGACATAA	121pb	XM_010792901.1	105	102.2	95.3	104.6
Ferritin middle chain (Fm) Rv	AACGCTACTCCACTTCCCCA						
Ferroportin (Fp) Fw	GCCATGGGTCACGTCATGTA	127pb	XM_010788639.1	97.4	107.2	96.6	92.7
Ferroportin (Fp) Rv	GTTAGACGGTGGTGGGAAGG						
Hepcidin type 2 (Hamp2) Fw	GAGCCGATGAGCGTTGAAAG	139pb	XM_010774309.1	105.9	105.9	94.9	99.9
Hepcidin type 2 (Hamp2) Rv	AGGACAATCCGCAGACACC						
Ceruloplasmin (Cp) Fw	GTTTCCAGCCACCTTTCAGACAGT	104pb	XM_010778042.1	109.6	98.7	100.9	101.3
Ceruloplasmin (Cp) Rv	TCGCCTCCATGCCACCTTTAAT						
Interleukin 6-receptor α chain (IL6Rα) Fw	TGGCAGCTTAAGCCAGAAGG	148pb	XM_010787044.1	109.7	97.6	106.3	96.7
Interleukin 6-receptor α chain (IL6Rα) Rv	GGCAATTGGAGCGTAGGTCT						
Interleukin 6-receptor β chain (IL6Rβ) Fw	GGACAATGAGATCGCCATGC	131pb	XM_010787241.1	100.7	98.3	101.5	100.8
Interleukin 6-receptor β chain (IL6Rβ) Rv	TGTCAACCCTTGATAAAGCCC						
Transferrin (Tf) Fw	CAGTGGTCAGGCGTTGAAGA	146pb	NM_001303296.1	107.3	105.9	99.1	95.8
Transferrin (Tf) Rv	TAGTAAGGCTCGGTGTGGGA						
Transferrin receptor type 1 (TfRC1) Fw	GTCCCCCAGAGAGAGTCCAT	127pb	XM_010774827.1	103.5	109.2	94.2	107.1
Transferrin receptor type 1 (TfRC1) Rv	TCGGAACAGGTTGAAGTCGG						
Housekeeping (18s) Fw	GTCCGGGAAACCAAAGTC	116pb	AF518193.1	107.9	101.6	108.6	99.7
Housekeeping (18s) Rv	TTGAGTCAAATTAAGCCGCA						

### Statistical Analysis

Data is shown as mean ± standard error (SE) of the mean. Normality and homogeneity of the variances were verified using the Shapiro-Wilk test. The logarithmic transformation was used if data did not follow a normal distribution. The Student’s *t*-test was used to compare the effect of LPS versus control for iron and gene expression data. The level of statistical significance was 5%. Because several comparisons did not achieve statistical significance and an increase in variability was observed in the LPS treated samples, a Principal Component Analysis (PCA) with rotation based on the Varimax equation with Kaiser Normalization was carried out order to determine the global sample distribution between control and LPS mean centered expression data in liver and head kidney of the two species. The software SPSS (IBM SPSS version 25) was used for the statistical analysis, applying univariate analysis of variance (ANOVA), means, box’s M and Fisher’s transformation to produce a correlation and covariance matrix for discriminant analysis. A Principal Component Regression based on a linear regression on principal components score was applied considering the component score coefficient matrix and component score covariance matrix to discriminate between control and LPS treatment groups. The RStudio software (version 1.0.143) was used to confirm PCA data and produce the confidence ellipses (95%), starting from centroid position for each tissue and species based on component regression of two dimensions (Principal components 1 and 2).

## Results

### Plasma Iron and Expression of Iron-Related Immune Genes

**FIGURE 1:**
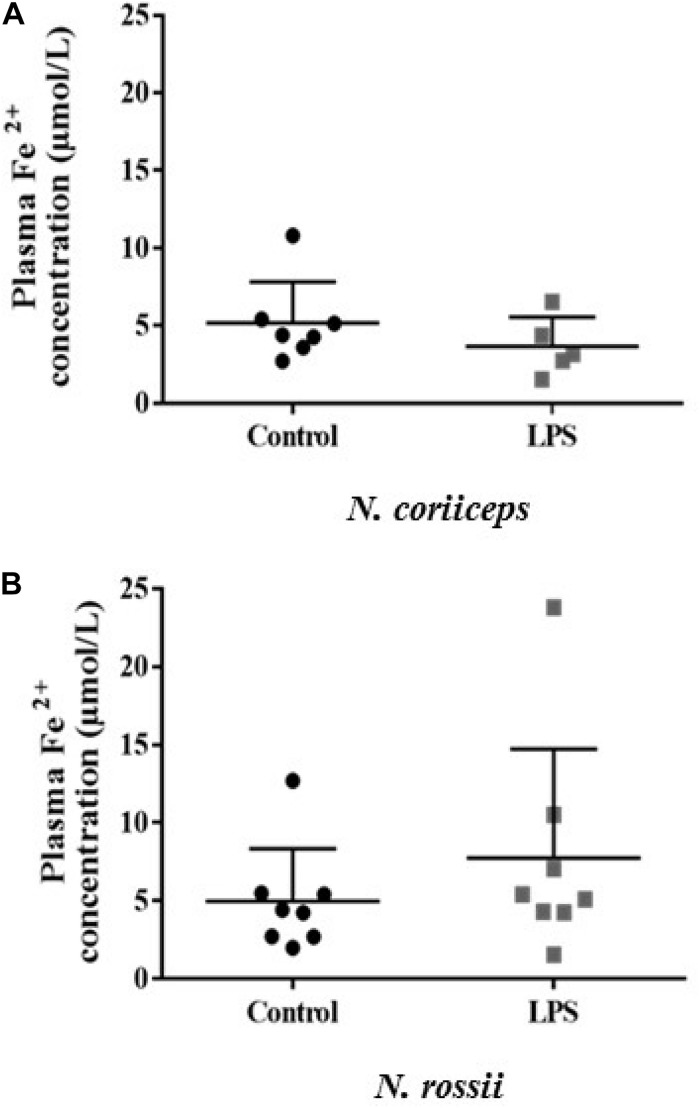
Iron concentration (Fe^2+^) in plasma at day 7 (5 days after last injection) in fish receiving two injections (on day 0 and day 2) of saline solution (Control) or LPS. **(A)** *N. coriiceps* (*n* = 7 and 5, respectively) **(B)** *N. rossii* (*n* = 8). The final concentration was calculated considering the dilution factor (9×) and molar mass (55.847 g mol^–1^). No statistical differences were observed among the different treatments (Student’s *t*-test, *P* > 0.05).

The concentration of plasma iron (Fe^2+^) varied between 1.53 and 10.8 μM in *N. coriiceps* and 1.55–23.8 μM in *N. rossii*, with no statistical difference between control and LPS treatment (Figures 1A,B).

**FIGURE 2:**
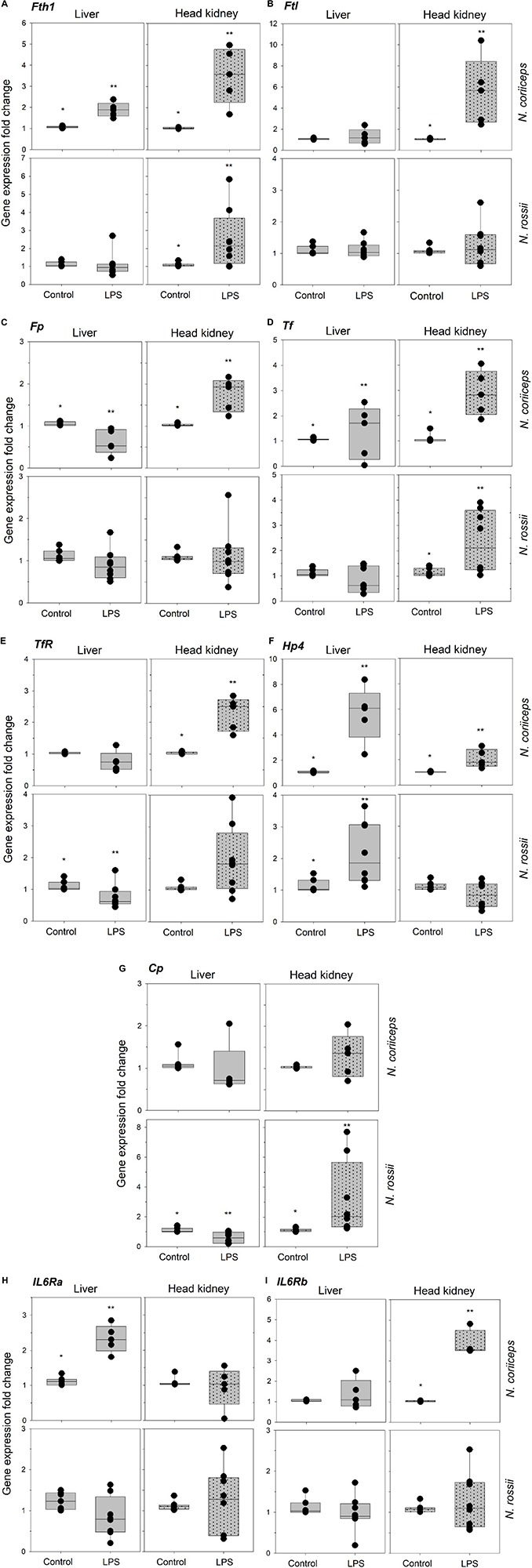
Gene expression of ferritin heavy chain (*Fth1*) **(A)**, ferritin middle chain (*Fm*) **(B)**, ferroportin (*Fp*) **(C),** transferrin (*Tf*) **(D)**, transferrin receptor 1(*TfRC1*) **(E)**, hepcidin 2 (*Hamp2*) **(F)**, ceruloplasmin (*Cp*) **(G)**, interleukin 6-receptor alpha chain (*IL6R*α) **(H)**, interleukin 6-receptor beta chain (*IL6R*β), **(I)** in liver and head-kidney of *N. coriiceps* (NC) and *N*. *rossii* (NR) injected with serum saline (Control) or LPS, on day 7 (5 days after last injection). Relative expression was calculated by the 2^–ΔΔCT^ method using the 18s ribosomal protein as the internal reference gene. Each gray bar value is the mean ± SEM and the black dot values correspond to each individual fish (*n* = 5–7 for *N. coriiceps* and *n* = 8 for *N. rossii*). Asterisks indicate statistical differences between control and LPS treated fish, for each tissue and species (Student’s *t*-test, *P* < 0.05).

The iron-related immune gene expression response to LPS varied with tissues and species. *N. coriiceps* was more responsive to LPS than *N. rossii*. In *N. coriiceps* of the nine genes analyzed, 4 were significantly upregulated (*p* < 0.05) in the liver and 7 in the head kidney, 3 of which were common to both tissues – *Fth1*, *Tf* and *Hamp2* (Figures 2A–I). In contrast, in *N. rossii* only *Hamp2* was significantly upregulated in the liver and 3 genes were significantly upregulated in head kidney – *Fth1*, *Tf* and *Cp* (Figures 2A–I). *Fp* was significantly downregulated in *N. coriiceps* liver while *Cp* and *TfRC1* were significantly downregulated in *N. rossii* liver (Figures 2C,E,G).

### Multivariate Analysis

Since the response to LPS was relatively weak but an increase in gene expression variability was apparent, even when not statistically significant, we sought to determine if there was an association of the gene expression responses with the experimental groups using a PCA for each tissue and species. In *N. coriiceps* liver, 78% of the gene expression variance was explained by the two first principal components (PC), 42% for PC1 and 36% for PC2. In head kidney, the two first PC explained 85% of the gene expression variance, 60.5% for PC1 and 25.2% for PC2. In *N. rossii* liver, 3 PC were required to explain almost 75% of the gene expression variance, 34.1% for PC1, 22.9% for PC2, and 18.3% for PC3. In head kidney, 3 PC explained 80% of the gene expression variance, 40.8% for PC1, 23% for PC2, and 15.7% for PC3.

From the component coefficient matrix and the rotated component matrix (Table 2), the differentiation between genes and treatment groups occurred mainly along PC1 for both tissues and species. However, the load factors of the different genes, regardless of treatment, vary widely for each PC, depending on tissue and species. The PCA analysis of gene expression in *N. coriiceps* liver shows that PC1 was strongly and positively correlated (>0.75) with *Fth1*, *Hamp2*, *IL6R*α and moderately (>0.5) with *Tf*, and inversely correlated with *Fp*, while PC2 directly correlates with *Cp*, *ILR6*β, *TfRC1*, *Fm* and less with *Fp*. In *N. coriiceps* head kidney, PC1 was strongly or moderately correlated (>0.75 or >0.5) to most genes tested with exception *IL6R*α, and PC2 correlated positively with *IL6R*α and less with *Cp*, and inversely with *Fm*. As indicated, above, in *N. rossii* dispersion was higher and three components were required to explain at least 75% of the distribution, but the same three genes appear to be responsible for determining the PC1 in both liver and head kidney. Thus, in liver, *Fm*, *Fp* and *ILR6*β were the most loading factors on PC1, *Fth1* and *TfRC1* on PC2, while PC3 was mostly positively correlated with *IL6R*α (>0.5) and inversely with *Hamp2*. In head kidney PC1 correlates strongly (>0.75) with *Fm*, *Fp* and *ILR6*β and moderately (>0.5) with *Fth1* and *Cp*. PC2 was mostly determined by *Tf* and *IL6R*α and PC3 directly by *Fth1* and *TfRC1* (>0.5) and inversely by *Hamp2* (<0.5).

**TABLE 2:** Principal Component Analysis by rotation method (Varimax with Kaiser Normalization) applied in different iron-related genes in liver and head kidney of *N. coriiceps* and *N. rossii*.

Rotated component matrix										

Tissue - Species	Liver – *N. coriiceps*		Head kidney – *N. coriiceps*		Liver – *N. rossii*			Head kidney – *N. rossii*		
				
Genes	Component		Component		Component			Component		
				
	1	2	1	2	1	2	3	1	2	3
Ferritin heavy chain (*Fth1*)	0.924	–0.66	0.972	–0.002	–0.078	0.861	–0.371	0.639	0.089	0.673
Ferritin middle chain (*Fm*)	0.228	0.847	0.689	–0.676	0.906	–0.117	0.027	0.985	–0.001	0.069
Ferroportin (*Fp*)	–0.766	0.589	0.917	–0.281	0.922	0.177	–0.036	0.951	0.071	–0.094
Transferrin (*Tf*)	0.547	0.428	0.966	0.030	0.089	0.737	0.172	0.035	0.928	–0.009
Transferrin receptor type 1 (*TfRC1*)	–0.428	0.865	0.930	–0.023	0.559	0.764	0.012	0.301	0.550	0.571
Hepcidin type 2 (*Hamp2*)	0.962	0.221	0.917	0.226	–0.225	0.217	–0.808	–0.208	0.349	–0.686
Ceruloplasmin (*Cp*)	–0.47	0.901	0.603	0.692	0.022	0.643	–0.113	0.546	0.490	0.169
Interleukin 6-receptor α chain (*IL6R*α)	0.968	–0.18	0.039	0.915	–0.023	0.619	0.674	–0.085	0.905	–0.069
Interleukin 6-receptor β chain (*IL6R*β)	0.424	0.847	0.875	–0.460	0.886	0.118	–0.062	0.972	–0.082	0.121
*Cumulative Variance Explained (%)*	*42.1*	*78.1*	*61.3*	*85.7*	*34.1*	*57.1*	*75.3*	*40.8*	*63.8*	*79.5*

**FIGURE 3:**
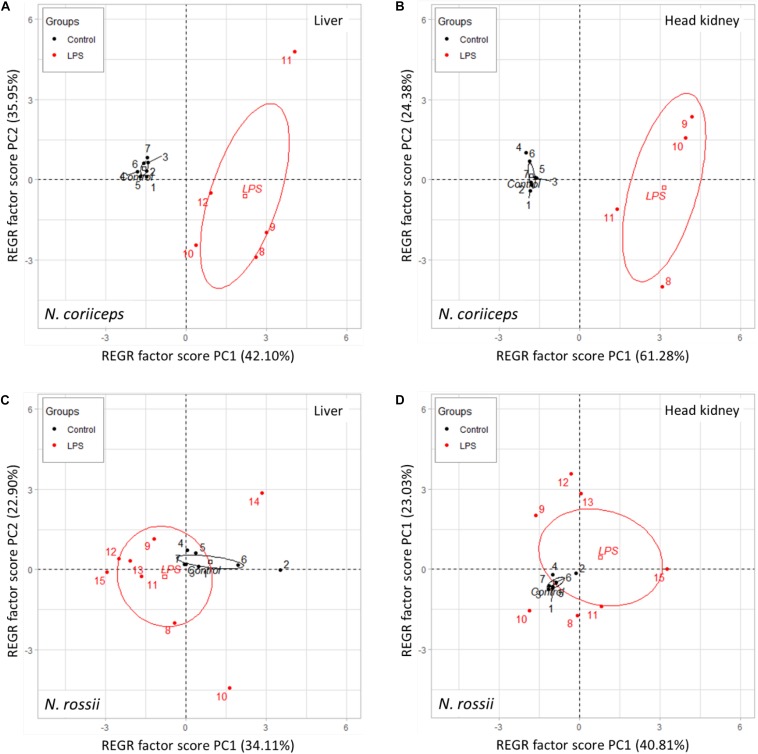
Scatterplot of principal component regression on two dimensions (PC1 and PC2) showing the confidence ellipses (95%) and respective centroids between control and LPS groups, in liver **(A)** and head kidney **(B)** of *N. coriiceps* and in liver **(C)** and head kidney **(D)** of *N. rossii.*

A Principal Component Regression used the first 2 PC variables as regressors to determine the variance between the group clusters. For both immune tissues of *N. coriiceps* the clusters of control and LPS groups aggregated wide apart and the variance of control individuals was low contrasting with the LPS treated fish which showed a much higher variance, spreading mostly along the PC2 axis (Figures 3A,B). In *N. rossii* the clusters of the control and LPS groups were closer together, including a slight overlapping, in both liver and head kidney expression distribution, with control centroids on the edge of the LPS 95% confidence ellipses. Nonetheless, and as for *N. coriiceps*, individuals of the control group clustered close together, as in *N. coriiceps*, while individuals of LPS group show high dispersion, along both PC1 and PC2 axis (Figures 3C,D). Overall, the analysis explains the allocation of all control individuals into the same treatment group for both species, whereas for LPS 82% of *N. coriiceps* and 85% of *N. rossii* were correctly classified as belonging to the same treatment group.

## Discussion

This study found that stimulation with lipopolysaccharide, a gram-negative cell wall component, had no effect on circulating Fe^2+^ levels, of two closely Notothenioid species, *N. coriiceps* and *N. rossii*, but caused modifications in the expression of iron metabolism related immune genes, both in liver and in head kidney. This gene response showed important differences in magnitude between species, and the effects of LPS were more attenuated and variable in *N. rossii* when compared to *N. coriiceps*.

In the present study no significant changes were observed in plasma Fe^2+^ between the two *Notothenia* species and in response to LPS. Since Fe^2+^ concentrations were within the range of measurements made in other teleosts (Congleton and Wagner, 1991; Welker et al., 2007; Kopp et al., 2011, 2014; Eslamloo et al., 2012; Hedayati et al., 2016), it suggests potentially similar requirements and homeostatic mechanisms between Antarctic and temperate fish. Although it has been shown in many vertebrate species that bacterial infection leads to a depletion of circulating iron (reviewed by Skaar, 2010; Johnson and Wessling-Resnick, 2012), most studies in fish showed either acute or week-lasting effects of LPS administration over the expression of iron-chelating or iron-transporting mechanisms but did not measure the ion itself (Prieto-Álamo et al., 2009; Liu et al., 2012; Martínez et al., 2017a) and thus the effectiveness and dynamics of LPS alone in lowering plasma iron is far from resolved. In rainbow trout, *Oncorhynchus mykiss*, LPS injection (1 mg/kg) caused a significant decrease in plasma iron within 48–72 h post-injection but circulating levels returned to normal by 96–120 h (Congleton and Wagner, 1991) and similar kinetics were observed in Atlantic salmon, *Salmo salar*, injected an identical dosage (Langston et al., 2001). It is therefore also possible that an effect in Fe^2+^ plasma concentrations may have been missed in our time-frame and dosage, which followed previous studies on temperate fish (Nayak et al., 2011; Guzman-Villanueva et al., 2014; Seppola et al., 2015; Chen et al., 2016) but may less adequate in these Antarctic species. In the present study fish were injected twice, at 0 and 48 h, and sampled at 120 h after the last injection, a design chosen taken in consideration that low temperature habitats may contribute to a slower innate immune system response, as it has been described in cold-water fish when compared to temperate fish (Tort et al., 2003; MacKenzie et al., 2008; Magnoni et al., 2015; Bonneaud et al., 2016; Chen et al., 2016; Abram et al., 2017; Martínez et al., 2017a).

The overall gene expression response, albeit not as vigorous as in other vertebrates (Collins, 2008; Johnson and Wessling-Resnick, 2012; Bethke et al., 2016), also suggests that an iron related immune response is active, much alike what it was seen in the sub-Antarctic Notothenioid *Eleginops maclovinus* (Martínez et al., 2017a), the Senegalese sole, *Solea senegalensis* (Prieto-Álamo et al., 2009) or the roughskin sculpin, *Trachidermus fasciatus* (Liu et al., 2012), among other fish species.

Several genes with important roles in iron metabolism responded in the liver and head kidney in one or both species. Interestingly, there were differences in responsiveness between the two species, with more genes responding and at a higher level in *N. coriiceps*. The reason for this is not immediately apparent but the two species, although captured in the same general area, tend to segregate with *N. rossii* in open and deeper channels and *N. coriiceps* usually under rocks in shallower waters (Jones et al., 2009; Kandalski et al., 2018; Barrera-Oro et al., 2019), which could lead to different exposure to potential pathogens and different immune responsiveness. Some *N. coriiceps* specimens can be found near the surface in low tide, hiding in rock crevices surrounded by algal debris, thus possibly more exposed to microorganisms. Additionally, while *N. coriiceps* lives in these coastal shallow waters throughout its life, in the case of *N. rossii* only the juveniles appear to use these areas, with adults moving into deeper and open waters (Jones et al., 2009), and a life stage difference can thus account for some of the variance in the responses.

The liver is a major organ for iron storage and the site of synthesis for many proteins involved in iron metabolism. Although we have not determined iron levels in liver, the overall changes in gene expression in both species seem to favor iron accumulation in liver cells although this is more evident in *N. coriiceps*. In *N. coriiceps* liver upregulation of *Fth1* and *Tf* may promote binding of intracellular iron while upregulation of *Hamp2* and downregulation of *Fp* should inhibit its export (Zahringer et al., 1976; Torti and Torti, 2002; Neves et al., 2009). In *N. rossii* liver the export machinery seemed to be downregulated (increased *Hamp2* and *Cp* and reduced *TfRC1* transcription) but the net effect is expected to be in the same direction and would reduce iron availability for extracellular bacteria.

The fish head kidney is involved in the immune response, but it is also the major haematopoietic organ, thus a likely responder to LPS and an important site for iron trafficking. In fact, expression of head kidney hepcidin genes has been described as a crucial regulator of erythropoiesis during anemia in fish (Joao et al., 2016). In our experiment, the tendency for intracellular accumulation of iron in the head kidney is apparent in *N. rossii* (by upregulation of *Tf*, *Fth1* and *Cp*) and also in *N. coriiceps* (upregulation of *Tf*, *Fth1, Fm*, and *Hamp2*) although in the later species iron turnover rate may be increased (by upregulation of *TfRC1* and *Cp*, possibly involved in extracellular transport). Overall this is consistent with previous reports which suggested an iron deficiency condition after LPS stimulation of the European sea bass, *Dicentrarchus labrax* (Neves et al., 2009) and the sub-Antarctic *Eleginops maclovinus* (Martínez et al., 2017a) and up-regulation of the same iron-related genes during experimentally induced anemia in fish (Joao et al., 2016).

Interleukins are modulators of haematopoiesis, inflammation and immune responses. It has been further shown in fish that IL-6 is induced in macrophages during sepsis, and suggested that this may concur to reduce iron availability by induction of hepcidin, as a means to limit the spread of infection (Costa et al., 2011). Interleukin-6 could not be analyzed despite several attempts to isolate the IL-6 sequence in both species, based on the *N. coriiceps* genome (available in NCBI database, under ID: GCF_000735185.1) (Shin et al., 2014) and in our transcriptomic data on *N. rossii* (unpublished). However, the two interleukin-6 receptor genes were analyzed, *IL-6R*α was highly expressed in liver and *IL-6R*β was highly expressed in head kidney of *N. coriiceps*. Interestingly, no significant change in gene expression response occurred in *N. rossii*. The differential response of IL-6R genes between the two species is consistent with iron metabolism-related genes response which was higher in *N. coriiceps*. This may suggest that also in these species IL-6 cytokines are involved in the induction of the synthesis of the iron regulatory hepcidin during hypoferremia inflammation (Berczi and Szentivanyi, 2003; Nemeth et al., 2004a; Shi and Camus, 2006; Roy et al., 2016). As indicated above, either the timing or a lower general immune response may explain the observations of low or poor immune responses. Although unlikely because of their close phylogeny, it is also possible that the two species may employ alternative pathways in iron metabolism defense. Indeed, species differences in physiology seems to exist. *N. coriiceps* appear to have lower thermal tolerance than *N. rossii* based on endocrine, metabolic and antioxidant parameters (Kandalski et al., 2018). Also, the more active *N. rossii* seems to have higher antioxidant defense system (ROS scavenge) enzyme activity than *N. coriiceps*, which could be related to their habits and different metabolic rates (Klein et al., 2017).

The PCA analysis also reflects the differences on the response among species, with a clearer separation between control and LPS-treated fish in *N. coriiceps* than in *N. rossii*. However, the large variability in responses between individuals, and specific genes in each tissue, especially in the LPS groups makes it difficult to identify which are the main genes reacting to challenge, and thus determining the different PC. These differences, however, show that overall, in both species, the weight of genes determining PC1 appears to be stronger for head kidney than for in liver, where PC2 has a relatively larger weight, which may show that more factors can be involved in the response in the latter tissue. Still, in both tissues of *N. coriiceps*, the PC1 is mostly determined by *Fth1* and *Hamp2*, while *Fm* and *Cp* appear relevant for PC2. In *N. rossii* PC1 in liver and head kidney is mainly dependent on the weight of *Fm*, *Fp* and also *IL6R*β, while the determination of PC2 is shared by more genes but with lower relative importance. Thus, it seems that iron-binding proteins and transmembrane iron-transporters are responding but that magnitude, time-frame and the repertoire of mechanisms vary widely between species and individuals upon challenge.

Indeed, regardless of the changes in the mean expression level of the genes tested, a striking effect of the LPS injection was the increase in the variability of the individual response, when compared to the fish injected with saline. This is observable when looking at the individual gene expression variation and becomes obvious when a PCA was performed. From the analysis it is obvious that some individuals clearly responded to LPS by mounting an immune/iron-related reaction, but not others, although control and LPS-treated fish generally group together in the respective 95% confidence cluster. This lack of synchronicity in response to a seemingly stressful process has been observed in many species, especially when using wild-caught specimens, with likely variations in their life-history experiences (Robertson et al., 2016), nutrition state (Ahola et al., 2017), physiological conditions (Killen et al., 2017) or stress response norms (Balasch and Tort, 2019). In our case, fish were acclimated before the experiments and treated equally thereafter, but changes in size, age and possibly on social condition (Filby et al., 2010) still existed. Additionally, this variability was also observed on the nature of the genes that responded and not only on the magnitude of the response. This could reflect differences in sensitivity or differences in baseline iron, resulting in an asynchronous response. As indicated above for plasma Fe^2+^ our initial assumption was that a slower metabolism at low temperature could delay the response to LPS compared to temperate teleosts, but this assumption may have been proven wrong. It is possible that an acute response to LPS may have been missed early in the relatively long timeframe of this experiment. Further studies, using several shorter time points, and including iron transport should help to understand innate nutritional immunity in Antarctic fish.

In summary, despite a less than robust immune response of Antarctic Notothenoids marked by considerable individual variability we have shown that overall LPS promotes the mobilization of genes important for iron retention in liver and head-kidney which should contribute to lower iron levels in extracellular fluids and fight bacterial infection through iron starvation.

## Data Availability Statement

The raw data supporting the conclusions of this article will be made available by the authors, without undue reservation, to any qualified researcher.

## Ethics Statement

The animal collection and experimentation were approved by the Portuguese Environment Agency, under the regulations set by the Treaty of Madrid for scientific investigation on Antarctica. The experiments performed were complied with the EU and Portuguese regulations for animal experimentation and the Guidelines of the Comisión Nacional de Investigación Cientifica y Tecnológica de Chile (CONICYT) and the Universidad Austral de Chile for the use of animals.

## Author Contributions

DM, CS, PG, AC, DP, JP, RO, and LV-C contributed to analyzing the data, working on the original manuscript and several revisions, and final version. PG, LV-C, and AC designed the experimental assay. CS and PG carried out the experiments and collected the samples. DM, CS, JP, and RO performed the lab analysis.

## Conflict of Interest

The authors declare that the research was conducted in the absence of any commercial or financial relationships that could be construed as a potential conflict of interest.

## Footnotes

1 https://www.ncbi.nlm.nih.gov/

2 https://www.ensembl.org/index.html

3 http://146.193.226.37/
