# Erratum: LPS Modulates the Expression of Iron-Related Immune Genes in Two Antarctic Notothenoids

**DOI:** 10.3389/fphys.2020.00262

**Published:** 2020-03-24

**Authors:** Frontiers Production Office

**Affiliations:** Frontiers Media SA, Lausanne, Switzerland

**Keywords:** *Notothenia coriiceps*, *Notothenia rossii*, iron metabolism, Antarctic fish, nutritional immunity

Due to a production error, the funder “Portuguese Foundation for Science and Technology (FCT),” “FCT-NSFC/0002/2016, PTDC/BIAANM/3484/2014, and CCMAR/Multi/04326/2019,” and a PhD fellowship “SFRH/BD/120040/2016” to “Carmen Sousa” was erroneously omitted.

The publisher apologizes for this mistake. The original article has been updated.

